# An enhanced method for sequence walking and paralog mining: TOPO^® ^Vector-Ligation PCR

**DOI:** 10.1186/1756-0500-3-61

**Published:** 2010-03-04

**Authors:** Benjamin B Orcheski, Thomas M Davis

**Affiliations:** 1 Genetics Graduate Program, University of New Hampshire, Durham, NH, 03824, USA; 2 Department of Biological Sciences, University of New Hampshire, Durham, NH, 03824, USA

## Abstract

**Background:**

Although technological advances allow for the economical acquisition of whole genome sequences, many organisms' genomes remain unsequenced, and fully sequenced genomes may contain gaps. Researchers reliant upon partial genomic or heterologous sequence information require methods for obtaining unknown sequences from loci of interest. Various PCR based techniques are available for sequence walking - i.e., the acquisition of unknown DNA sequence adjacent to known sequence. Many such methods require rigid, elaborate protocols and/or impose narrowly confined options in the choice of restriction enzymes for necessary genomic digests. We describe a new method, TOPO^® ^Vector-Ligation PCR (or TVL-PCR) that innovatively integrates available tools and familiar concepts to offer advantages as a means of both targeted sequence walking and paralog mining.

**Findings:**

TVL-PCR exploits the ligation efficiency of the pCR^®^4-TOPO^® ^(Invitrogen, Carlsbad, California) vector system to capture fragments of unknown sequence by creating chimeric molecules containing defined priming sites at both ends. Initially, restriction enzyme-digested genomic DNA is end-repaired to create 3' adenosine overhangs and is then ligated to pCR4-TOPO vectors. The ligation product pool is used directly as a template for nested PCR, using specific primers to target orthologous sequences, or degenerate primers to enable capture of paralogous gene family members. We demonstrated the efficacy of this method by capturing entire coding and partial promoter sequences of several strawberry Superman-like genes.

**Conclusions:**

TVL-PCR is a convenient and efficient method for DNA sequence walking and paralog mining that is applicable to any organism for which relevant DNA sequence is available as a basis for primer design.

## Background

Efforts to obtain desired gene and promoter sequences often rely on exploitation of fragmentary genomic or cDNA sequence information available from homologous or heterologous sources as a basis for PCR primer design. Various PCR-based sequence walking techniques have been developed for acquiring previously unknown genomic sequence flanking a known site [1-5]. All such techniques share a common strategy: creation of a distal priming site or sites for use in conjunction with priming sites in known genomic sequence. However, these techniques vary in how the distal priming site is created.

**Figure 1:**
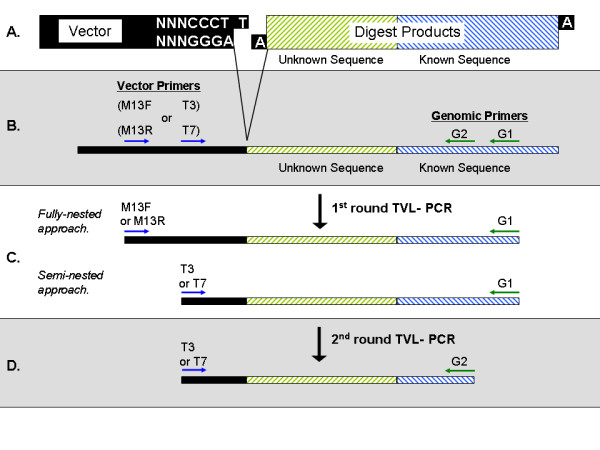
**Overview of TVL-PCR**. **A**. A pool of chimeric templates is generated using a ligation reaction that joins end-repaired genomic restriction fragments to TOPO vectors. Ligation is required only between one end of each genomic fragment and a vector molecule. **B**. A chimeric template molecule contains two priming sites (G1 and G2) within the genomic fragment and two appropriately oriented priming sites (M13F and T3, or M13R and T7) within the conjoined vector. **C**. In the first round of TVL-PCR, the G1 genomic primer is paired with a vector primer. In the fully nested approach an M13_ primer is used. Because the orientation of ligation is unknown, the G1 primer must be paired in separate reactions with M13F and with M13R. In the semi-nested approach, the G1 primer is paired with a T_ primer. **D**. In the second round of TVL-PCR, the G2 genomic primer is paired with either the T3 or the T7 primer, as appropriate. The T3 primer must be used if either the M13F or T3 primer was previously used in the first round of TVL-PCR, while the T7 primer must be used if either the M13R or T7 primer was previously used.

Our method employs the pCR^®^4-TOPO^® ^(Invitrogen, Carlsbad, California) as a linker, thereby combining the advantages of T/A ligation [2] with those of using a vector as linker [1,3]. In our approach, called "TOPO^® ^Vector-Ligation PCR" (TVL-PCR), the pCR4-TOPO vector is employed as linker. Briefly, genomic DNA of interest is subjected to restriction digestion using any restriction enzyme that produces either blunt or recessed 3' ends. Digestion is followed by end-repair, producing a pool of fragments with single, 3' adenosine overhangs at each end. These fragments are shotgun ligated to pCR4-TOPO vector (Figure 1A), creating chimeric molecules containing known priming sites at both ends (Figure 1B). The ligation product pool serves as template for the initial round of TVL-PCR (Figure 1C), and this product is used as template in the fully- or semi-nested second round of TVL-PCR (Figure 1D). The final product of TVL-PCR spans a segment of known sequence, an adjacent segment of the unknown sequence of interest, and a short vector segment.

We describe TVL-PCR and explain how we used it to obtain full length sequence and partial promoter sequence of several strawberry Superman-like genes. We also successfully used TVL-PCR for paralog mining (results not shown), which is the amplification of multiple members of a gene family using degenerate primers based on conserved sequences as priming sites.

## Materials and methods

### Template Preparation for TVL-PCR

DNA was isolated from unexpanded leaf tissue of *Fragaria virginiana *accession L2 (CFRA 1995) as described [6], except that no chloroform:octanol solution was included in the microfuge tube to which CTAB slurry was transferred. Per reaction, 400 ng of genomic DNA was digested with 20 U of *Eco*RI, *Bam*HI, or *Hin*dIII (New England Biolabs, Ipswich, Massachusetts) in a 40 μl reaction that was incubated overnight at 37°C. Employed restriction enzymes must produce recessed 3' (5' overhangs) or blunt ends. Digestion was verified by electrophoresis of 100 ng digested genomic DNA and undigested comparator on a 1% agarose TBE gel.

### Repair of Fragment Ends and Addition of 3'A Overhang

End-repair employed 20 μl (200 ng) of each digested DNA sample with 1 μl of 10 mM dNTP mix, 1.3 μl sterile water, 2.5 μl of EconoTaq^® ^buffer (Lucigen, Middleton, Wisconsin) and 0.2 μl (1 U) EconoTaq DNA polymerase. Reactions were incubated at 72°C for 30 minutes to fill in recessed 3' ends of cut sites, add a 3' adenosine overhang, and heat-inactivate the restriction enzyme.

### Ligation

End-repaired DNA was ligated to the pCR4-TOPO vector (Invitrogen) using 4.5 μl (36 ng) end-repaired DNA solution, with 0.5 μl (5 ng) of TOPO vector and 1 μl of supplied salt solution. The reaction was gently mixed and incubated at room temperature for one hour.

### Primer Design

The design of the gene-specific primers relied on alignments comprised of Superman-like sequences from four heterologous sources (*Petunia*, *Nicotiana*, *Arabidopsis*, and *Malus*), and strawberry Superman-like sequences obtained via conventional PCR amplification using degenerate primers targeted to sites conserved among the four heterologous sequences (results not shown). Two strategies were employed. For paralog mining of new Superman-like genes, degenerate primers were targeted to genomic sites identified as conserved among the heterologous and strawberry sequences. For sequence-specific walking to extend initially obtained gene segments, sequence-specific primers were targeted to genomic sites that were *not conserved *among the aligned heterologous and strawberry sequences. Genomic primers were always sited at least 50 bases upstream of the transition point from known to unknown sequence, to provide a sufficient read of known sequence in the resulting TVL-PCR product to confirm sequence identity and continuity.

### TVL-PCR

**Figure 2:**
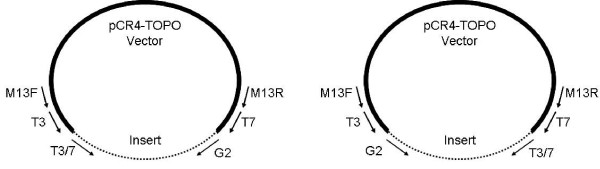
**Recombinant vector with primer sites and alternate insert orientations**. For sequencing, the products generated by TVL-PCR must be cloned into the TOPO vector. Two possible insert orientations are possible, as shown. M13F or M13R primers must be used for sequencing because they provide unique priming sites in the recombinant plasmid. The T3 or T7 primers are unsuitable for sequencing because they are represented in both the vector and the insert.

The thermocycler profile used for both the first and second rounds of TVL-PCR was: initial denaturation at 94°C for 1 min; then 35 cycles of 94°C for 30 sec, 52°C for 30 sec, 68°C for 4 min; followed by a final extension at 68°C for 10 minutes. For each ligated template, two first-round amplification reactions were employed for the initial step of TVL-PCR, because the genomic fragment can ligate to the vector in alternate orientations (Figure 2). In one first-round TVL-PCR reaction (Figure 1C), the distal genomic primer (G1) was paired with the M13F or T3 vector primer, while in the other the G1 primer was paired with the M13R or T7 vector primer. In the first round, use of a distal M13_ (M13F or M13R) vector primer allows for subsequent vector primer nesting, while use of a proximal T_ (T3 or T7) vector primer precludes subsequent vector primer nesting (and is therefore recommended only if incompatibility exists between M13 primers and the distal genomic primer). For the second-round of TVL-PCR (Figure 1D), each first-round product was used as a template, and the nested genomic primer (G2) was paired with the appropriate T_ vector primer (T3 or T7, respectively - see below).

The 25 μl first-round TVL-PCR reactions contained 3 μl ligation reaction template, 0.5 μl of 20 μM G1 genomic primer, 0.5 μl vector primer, 0.1 μl (0.5 U) AccuPrime™ Taq DNA Polymerase High Fidelity (Invitrogen), and 2.5 μl 10× AccuPrime PCR buffer II. The first-round product (10 μl) was visualized on a 2% agarose 1% TBE gel run at 4.1 V/cm for 80 minutes. The second-round of TVL-PCR was performed with 1 μl of first-round product as the template, and 0.5 μl of 20 μM stock of both the nested G2 genomic primer and the appropriate vector primer. If the first-round vector primer was M13F or T3, then T3 was used as the second-round vector primer. If the first-round vector primer was M13R or T7, then T7 was used as the second-round vector primer.

### Cloning and Sequencing of TVL-PCR Products

The amplification products from each second round TVL-PCR reaction were cloned using the Invitrogen TOPO TA Cloning^® ^kit for sequencing, per the manufacturer's instructions. Transformed cells were plated on LB agar plates containing 50 μg/ml ampicillin and were grown at 37°C overnight. Colony PCR was performed using M13 primers provided with the TOPO cloning kit to confirm insert size. Plasmids were isolated from clones of interest using the Promega Wizard^® ^*Plus *SV Minipreps DNA Purification System (Promega, Madison, WI). The plasmid inserts were then sequenced bidirectionally on an Applied Biosystems 3130 Genetic Analyzer (Applied Biosystems, Foster City, CA), using M13 forward and M13 reverse sequencing primers. The T3 and T7 sequencing primers cannot be used for this purpose, because their priming sites may be represented twice in the final TOPO clone: once in the cloning vector itself and once in the cloned product of second round TVL-PCR (Figure 2).

## Results

### Paralog Mining

Use of degenerate primers targeted to genomic sites conserved among heterologous Superman sequences resulted in acquisition of multiple candidate clones from strawberry accession L2 (results not shown), including that of clone 143-2-1. The latter sequence provided the basis for design of site-specific primers used for sequence walking in accession L2, as described below.

### Sequence Walking

**Figure 3:**
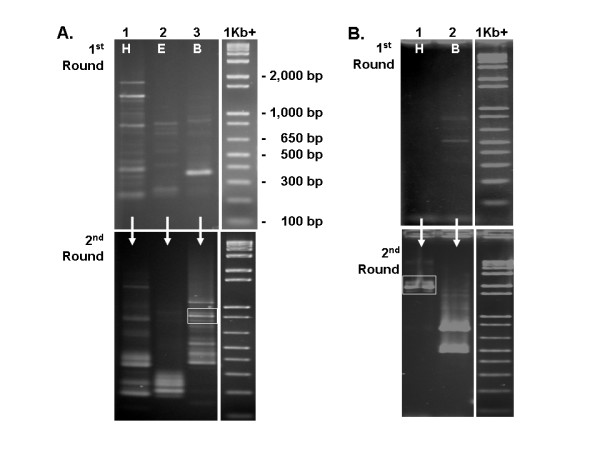
**Extension of sequence from clone 143-2-1 in the 5' (A) and 3' (B) directions**. First round TVL-PCR products (top) were generated from templates prepared using *Hin*dIII (H), *Eco*RI (E), or *Bam*HI (B) digestion of genomic DNA. First round products were used as templates to generate second round products (bottom). Boxes (bottom gels) indicate the locations of the second round TVL-PCR products that proved to be the desired products.

Specific primers targeted to non-conserved sites in clone 143-2-1 were designed (Table 1) and used to extend this gene fragment in both the 5' and 3' directions. For walking in the 5' direction, G1 primer 14321 5'F1 paired with the M13R vector primer produced a spectrum of products in the first-round reaction using *Hin*dIII (H), *Eco*RI (E), or *Bam*HI (B) digested genomic DNA as template (Figure 3A - top). The second round of TVL-PCR, using the first-round products as template with nested G2 primer 14321 5'F2 and the nested T3 vector primer, produced a diminished spectrum of bands in the H and E lanes, but a proliferation of bands in the B lane (Figure 3A - bottom). After shotgun cloning of the second round TVL-PCR products, colony PCR, and sequencing of three clones selected on the basis of favorable size (> 500 bp), one clone containing a Superman-like sequence was found. Its size corresponded to the boxed band in Figure 3A (bottom). This newly acquired sequence extended upstream from the known gene sequence to the putative start codon and beyond.

**Table 1:** Primer Information.

Primer Name	Primer Sequence	Tm (°C) *
Vector Primers		
M13F	5'-GTAAAACGACGGCCAG-3'	50.7
M13R	5'-CAGGAAACAGCTATGAC-3'	47.0
T3	5'-ATTAACCCTCACTAAAGGGA-3'	50.3
T7	5'-TAATACGACTCACTATAGGG-3'	47.5
Gene Primers		
14321 5' F1	5'-AGGGTTAGGTTTAAGGTTGAG-3'	51.8
14321 5' F2	5'-GTGAGGGTACGAAAGTAGG-3'	51.9
14321 3' F1	5'-TCGCAAGTTGAACTATGTATCC-3'	52.6
14321 3' F2	5'-CAGTTTGTTCAGGACTGAGT-3'	52.2

Vector primer sequences are standard, and are provided here for convenience. Gene primers were designed by us.

*Melting temperature values were determined by the Integrated DNA Technologies OligoAnalyzer application [7], and may not be the same as those published elsewhere for M13_ and T_ primers.

For walking in the 3' direction, the first round of TVL-PCR employed G1 primer 14321 3'F1 paired only with the T3 vector primer. The product from this reaction (Figure 3B - top) was used as template for the second round of TVL-PCR using the nested G2 primer 14321 3'F2 in conjunction with the T3 vector primer. Shotgun cloning of the second round TVL-PCR products (Figure 3B - bottom), followed by colony PCR and sequencing of two appropriately sized products (> 500 bp), yielded one product that contained the targeted sequence. The respective PCR product size corresponded to the boxed gel band (Figure 3B - bottom). This product was slightly less than 2 kb in length, and extended well into the 3' UTR of the targeted gene.

Overall, a total of five clones were sequenced from shotgun cloned and size-selected TVL-PCR products, of which one provided targeted sequence extension in the 5' direction and another in the 3' direction. The remaining three sequenced clones were not the targeted sequence, but displayed a vector primer sequence at one end and a respective genomic primer sequence at the other, indicating that each arose from non-specific priming by the genomic primer.

Using methods similar to those described above, TVL-PCR was also used successfully to extend the sequence of Superman-like clone 7266 upstream far enough to surpass the putative start codon (results not shown). In addition, the use of degenerate primers resulted in the acquisition of 5' and 3' sequences from three additional Superman family sequences in strawberry. All strawberry Superman-like genes isolated from chromosome walking via TVL-PCR have been deposited in GenBank under accession numbers, GU830924: 7266 5' walk, GU830926: 14321 5' walk, and GU830925: 14321 3' walk.

## Discussion

TVL-PCR adds a useful new option to the toolkit of methodological choices for sequence walking. By exploiting T/A ligation by using the pCR4-TOPO vector as a linker, TVL-PCR expands the spectrum of restriction enzymes employable for digestion of genomic DNA by eliminating dependence on corresponding restriction sites in the vector linker as encountered in Single Specific Primer PCR [1] and Rapid Amplification of Genomic DNA Ends [3].

Beneficially, methods that employ T/A ligation, including TVL-PCR and T-Linker PCR [2], preclude the possibility of genomic and/or vector fragment re-ligation to each other, or self-ligation (i.e., circularization). Because genomic DNA fragments with 3' adenosine overhangs can only ligate to a molecule having a 3' thymidine overhang, chimeric ligation constructs comprised of multiple genomic fragments are also precluded.

With ligation-dependent methods, duplicate linker ligation at both ends of a genomic fragment would create opportunity for "single primer amplification" via linker priming at both ends. The TVL-PCR procedure minimizes this problem by maintaining a high genomic DNA to vector ratio, minimizing the number of genomic fragments that acquire ligated vector molecules at both ends. We used 36 ng of genomic DNA in the ligation reaction, which after complete digestion with a six-base restriction enzyme should result in 8.2 billion fragments present in the ligation reaction. At a concentration of 10 ng/μl, 0.5 μl of TOPO vector yields 1.2 billion molecules, and thus roughly a ratio of seven genomic DNA fragments to one vector molecule. In various applications of the TVL-PCR technique, we have yet to encounter a product primed by a vector primer at both ends.

## Conclusions

TVL-PCR is an efficient and effective method for genomic sequence walking. It offers significant advantages, allowing choice among a large selection of restriction enzymes, and requiring only small amounts of template DNA. Using TVL-PCR, we have isolated entire coding and partial promoter sequences of several Superman-like genes from strawberry.

## Competing interests

The authors declare that they have no competing interests.

## Authors' contributions

BBO conceived and developed the technique, and performed the research. TMD provided technical support, helped draft the manuscript, and contributed to the design and trouble shooting of the procedure. Both authors read and approved the final manuscript.
